# Norovirus interactions with the commensal microbiota

**DOI:** 10.1371/journal.ppat.1007183

**Published:** 2018-09-06

**Authors:** Meagan E. Sullender, Megan T. Baldridge

**Affiliations:** Division of Infectious Diseases, Department of Medicine, Edison Family Center for Genome Sciences and Systems Biology, Washington University School of Medicine, St. Louis, Missouri, United States of America; University of Florida, UNITED STATES

## Introduction

**Fig 1:**
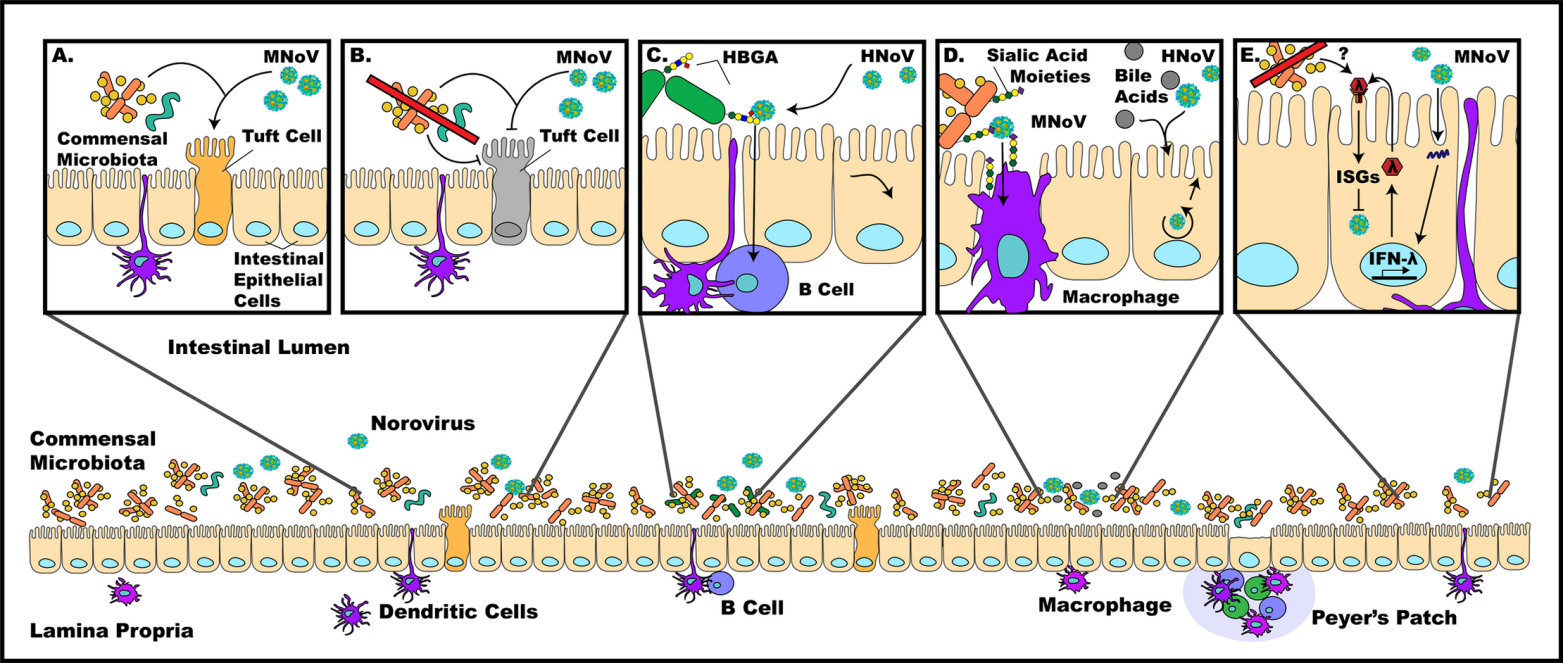
Norovirus pathogenesis is affected by many factors in the enteric environment. a) The presence of commensal bacteria allows for efficient MNoV infection, with tuft cells being one rare cell population infected. b) Absence of commensal bacteria reduces MNoV titers by depleting tuft cell populations and potentially altering innate immune responses during persistent MNoV infection. c) Binding of HNoV to HBGA-positive enteric bacteria has been found to facilitate infection of B cells. d) Sialic acid moieties on the cell surface have been found to act as coreceptors for MNoV infection of macrophages, while bile acids have been found to be important for the establishment of HNoV infection of enterocytes. e) MNoV infection has been found to trigger the expression of IFN-λ in infected cells, up-regulating interferon simulated genes that restrict viral replication and subsequent spread of infection—the mechanism of this process has yet to be characterized, but commensals are believed to play a major regulatory role. HBGA, histo-blood group antigen; HNoV, human norovirus; IFN-λ, interferon lambda; MNoV, murine norovirus.

Human norovirus (HNoV) is the leading cause of epidemic nonbacterial gastroenteritis worldwide, causing an acute diarrheal infection and occasionally chronic infection in immunocompromised individuals. Mouse and tissue culture models utilizing murine norovirus (MNoV) have allowed for interrogation of viral mechanisms of infection and pathogenesis. Here, we outline the interactions between the commensal microbiota of the intestine and norovirus and their implications (Fig 1).

## Question 1: Is norovirus infection in mice a good model for norovirus infection in humans?

Due to the fact that HNoV cannot readily grow in mice and, until recently, has not been culturable in vitro, the use of MNoV has provided robust animal and tissue culture model systems, which allow for mechanistic studies of an orthologous pathogen [1–4]. The MNoV model system allows for the merging of basic mechanistic principles of infection and replication from cell culture systems to pathogenesis in a host system that is both genetically malleable and affordable.

Thus far, MNoV studies have allowed for the elucidation of a species-specific proteinaceous receptor (CD300LF) and viral tropism for a rare intestinal epithelial cell population called tuft cells during persistent infection as well as macrophages, dendritic cells, and lymphocytes during acute infection in vivo [5–7]. Many external factors outside of the narrow window of host cell–virus interactions that affect NoV pathogenicity have also been identified, such as bile, sialic acid, and intestinal bacteria [2,5,6,8]. Additionally, host mucosal cytokine interferon lambda (IFN-λ) has recently been identified as a potent anti-NoV molecule, opening the possibility of its therapeutic use for HNoV in the future [9]. Multiple strains of MNoV allow for the study of both chronic (MNV.CR6, MNV-3) and acute (MNV-1, MNV-1.CW3) infection, further adding to the strengths and complexity of this model system [10].

Despite some differences in symptom presentation and species-specific receptors, HNoV and MNoV exhibit many similarities in cellular tropism, requirement of carbohydrate attachment factors, and potential for persistent viral shedding after symptom resolution [3,10]. However, some clinical symptoms do differ between MNoV and HNoV infections, most notably the lack of vomiting and inability to produce more than statistically significant mild diarrhea in mice [11].

## Question 2: Do all strains of norovirus depend on the commensal microbiota for infection?

A large and diverse population of commensal microbes, consisting of bacteria, viruses, fungi, and parasites, reside within the intestinal lumen. NoV, being an enteric pathogen, encounters and interacts with members of this community, resulting in outcomes beneficial or detrimental to the host. HNoV has been found to interact directly with commensal (*Enterobacter cloacae*) and pathogenic (*Clostridium difficile*) bacterial species via the viral capsid and histo-blood group antigen (HGBA)-like carbohydrates expressed on bacterial surface membranes [12,13]. In addition, both HNoV and MNoV have been reported to bind sialic acid residues, which can be expressed on bacteria, suggesting that MNoV could also interact directly with the enteric microbiota [4,14].

Experimental alteration of the enteric microbiota with oral antibiotics drastically depletes the intestinal bacterial population. This in turn reduces the severity of acute MNoV infection (reduced MNV-1 titers) and also prevents or reduces persistent infections (drastically reduced MNV.CR6 and MNV-3 titers) in the ileum and colon [15]. Additionally, infection by MNV.CR6 can be rescued by fecal microbiota transplant (FMT) from nonantibiotic-treated to antibiotic-treated mice, highlighting the importance of commensal bacteria for MNoV infection [15]. While all murine NoV strains tested and reported thus far exhibit a dependence upon commensal bacteria for infection, further studies will be needed to determine whether this is a phenomenon affecting all HNoV and MNoV strains and to define strain-specific mechanisms.

One putative explanation by which bacteria can promote MNoV infection comes from the recent description of tuft cells as the physiologic target cell of persistent MNoV infection and propagation. Within the mouse intestine, tuft cells are regulated by commensal bacteria such that antibiotic treatment correlates with reduced numbers of tuft cells and leads to reduced viral titers [6]. In addition to commensal bacteria, parasitic worms (such as *Trichinella spiralis*) also exhibit a proviral effect in the context of MNoV infection [16,17] via induction of tuft cells by type 2 immune responses (IL-4, IL-25 cytokines) [6]. Thus, both bacteria and enteric metazoans can regulate NoV infection.

In addition to microbe–NoV and microbe–host interactions impacting viral pathogenesis, NoV infection itself can also alter the enteric microbial communities of the host. This virus-induced dysbiosis is characterized by an enhanced *Firmicutes* to *Bacteroidetes* ratio. This alteration is seen both in a subset of HNoV infections and early acute MNoV infections (MNV-1) [18,19]. However, this effect was not detected in longitudinal studies of both acute and persistent strains of MNoV (MNV-1, MNV.CR6, and MNV-4), suggesting potential temporal or facility-based effects [20].

## Question 3: How do commensal bacteria regulate enteric virus infections via immune skewing?

The host intestinal immune system is highly regulated by a complex interplay of various lymphoid tissues, immune cells, cytokines, and their receptors [21–23] and possesses three distinct layers: mucus, epithelia, and lamina propria. In the small intestine, mucus-secreting goblet cells and antimicrobial peptide-secreting Paneth cells form the mucosal barrier that segregates commensal bacteria from the intestinal epithelia [24]. Intestinal epithelial cells directly interact with and survey the gut environment in coordination with innate lymphoid cells, which communicate with the immune system via secretion of cytokines and chemokines [24–26]. Dendritic cells ferry antigen from the lumen across the epithelial barrier to draining lymph nodes and mucosal lymphoid tissues in the lamina propria [27], and innate inflammatory signals and other luminal signals activate T- and B-cell responses [26]. These interacting layers play a large role in maintaining the microbiota and host immune system in homeostasis as well as regulating infection, inflammation, and autoimmunity.

While the interactions between commensal bacteria, enteric viruses, and the intestinal immune system are still poorly understood, several recent studies have suggested important interplay between these factors. Innate immune responses are primed via commensal bacterial recognition by the enteric epithelium, which activates antiviral intestinal responses after a secondary viral-induced signal within the gut [22]. In contrast, mouse mammary tumor virus (MMTV) has evolved to evade innate immune responses by binding bacterial lipopolysaccharide, inducing the immunosuppressive cytokine IL-10 via Toll-like receptor signaling pathways [21]. This effect is entirely dependent upon the microbiota; mice receiving parenteral administration of MMTV do not experience a suppressed immune response, and antibiotic-treated or germ-free mice receiving MMTV orally fail to pass MMTV to their offspring. Microbial modulation of innate immunity also offers a second explanation of the preventive effect of antibiotics on MNoV infection: it is the result of the bacterial microbiota hindering a yet-to-be-identified immune pathway, which limits the antiviral efficacy of IFN-λ during persistent MNoV infection [23]. Evidence for this comes from experiments demonstrating that mice lacking IFN-λ signaling no longer require commensal bacteria for successful MNoV infection [23]. This triangle of interactions—commensals, viral pathogen, and host—produce anti- or proviral environments through many disparate and yet-to-be-characterized mechanisms.

## Question 4: Does a dependence on the commensal microbiota apply to other viruses?

Other enteric viruses, including rotavirus and poliovirus, have been found to depend on enteric bacteria to infect, similar to MNoV [28,29]. Commensal bacteria act as a proviral factor during poliovirus infection, as antibiotic treatment results in mice being less susceptible to infection and a reduced viral load in the intestine [28]. The mechanism underlying this involves viral particles binding to bacterial lipopolysaccharide, causing enhanced host cell receptor binding and virion stability [28,30]. Paradoxically, microbial depletion was found to increase antibody responses against rotavirus, which may contribute to enhanced viral clearance during antibiotic treatment [29].

In contrast, nonenteric viral infections are enhanced in mice depleted of commensal bacteria. For neurotropic flavivirus (West Nile, dengue, Zika) infections, depletion of the enteric microbiota significantly increased viral susceptibility, viral burden, disease severity, and lethal outcomes in mice [31]. Additionally, respiratory influenza A virus (IAV) and lymphocytic choriomeningitis virus (LCMV) infection have been found to be intensified (sustained, high viral titers in lung tissue and serum, respectively) due to impaired immune responses secondary to depletion of gram-positive bacteria in the gut [32,33].

In these cases, antibiotic treatment reduced virus-specific cluster of differentiation 8+ (CD8+) T-cell responses, though there is apparent variation in the manifestation of defects. Antibiotic treatment resulted in decreased numbers of dendritic cells (DCs) for antigen presentation in the case of flavivirus infection, whereas in the case of IAV and LCMV, there was a defect in DC migration to lymph nodes attributed to reduced inflammasome activation upon infection [31,32]. The bacterial metabolite desminotyrosine was also found to regulate type I IFN signaling in the lung to control IAV infection [32,34]. These findings suggest that bacteria interact with both innate and adaptive immune systems to control both local and systemic antiviral responses, leading to distinct outcomes for enteric and nonenteric viruses.

## Question 5: Does microbiome modulation have therapeutic potential for infectious diseases in humans?

While it is clear that the microbiome plays a significant role in both infectious and noninfectious diseases alike, much remains unknown about the exact mechanisms of action. FMTs have proven to be an effective treatment for *Clostridium difficile* (*C*. *diff*) infection and treatment-resistant irritable bowel syndrome (IBS) and may have potential in inflammatory bowel diseases [35]. It is likely that different underlying mechanisms contribute to the efficacy of these treatments; for example, specific bile acids regulated by intestinal bacteria are critical for resistance to *C*. *diff* infection [36,37]. Targeted administration of efficacious microbes would be ideal to prevent disease, and we are just beginning to identify the specific bacterial species that may regulate diseases from multiple sclerosis to diabetes to norovirus.

While it may appear that treating HNoV patients with antibiotics would prove beneficial since antibiotic treatment reduces MNoV titers in mice, it may actually cause more harm than good due to the overall beneficial impact of the microbiome on human health and the potential for increased susceptibility to other viral, fungal, or bacterial infections. Probiotics may be a better therapeutic option, such as adding helpful microbes to a patient’s microbiome to fight infection or as a form of biological vaccine adjuvant. And in the case of NoV, development of drugs that temporarily mitigate the effect of commensal microbes and their metabolites without clearing bacterial populations could prove to be a viable treatment option in the future.
